# UNDERUTILIZATION OF TRANSITIONAL CARE MANAGEMENT SERVICE AMONG MEDICARE BENEFICIARIES AT HIGH RISK OF READMISSION

**DOI:** 10.1093/geroni/igad104.3787

**Published:** 2023-12-21

**Authors:** Chan Mi Park, Ellen McCarthy, Sandra Shi, Brianne Olivieri-Mui, Jieun Jang, Dae Hyun Kim

**Affiliations:** Hebrew Senior Life, Boston, Massachusetts, United States; Hebrew Senior Life, Boston, Massachusetts, United States; Hebrew Senior Life, Boston, Massachusetts, United States; Northeastern University, Boston, Massachusetts, United States; Hebrew Senior Life, Boston, Massachusetts, United States; Harvard Medical School, Boston, Massachusetts, United States

## Abstract

The CMS introduced transitional care management (TCM) service in 2013 to improve care transitions from hospital to home and reduce readmission. TCM uptake has been slowly increasing over time; however, it remains uncertain whether TCM is delivered to patients at the highest risk of readmission. We used a 5% random sample of 2015-2019 Medicare fee-for-service claims. TCM service was identified using CPT codes and demographic information, census region, diagnosis codes, and frailty were measured. Of 1,556,347 eligible discharges (mean [standard deviation] age, 78.3 [7.8] years, 56.7% female, 84.2% White), TCM was delivered in 169,536 (10.9%) discharges and 30-day readmission occurred in 123,796 (8.0%) discharges. The 30-day readmission risk was higher among beneficiaries who were < 75 years (8.1%) vs ≥75 years (7.9%), male (8.7%) vs female (7.4%), Black (9.3%) or Hispanic (8.7%) vs White (7.8%) and Asian or Pacific Islander (8.0%), with dementia diagnosis (8.2%) vs without (7.9%), or with greater frailty (robust to severe frailty: 6.1% to 14.1%). TCM use was not necessarily higher among those with high-risk characteristics. Notably, TCM was more likely to be delivered to beneficiaries who were ≥75 years (12.3%) vs < 75 years (8.8%), White (11.3%) and Asian or Pacific Islander (10.8%) vs Black (9.0%) and Hispanic (8.4%), without dementia (11.0%) vs with (10.6%), and pre-frail or mildly frail (11.4%) vs moderately or severely frail (10.0-10.5%). Our study found that TCM is not delivered effectively to Medicare beneficiaries at high risk of readmission, including racial and ethnic minority groups, and those with moderate-to-severe frailty.

